# The trigger-timing framework for long COVID: using symptom triggers and temporal patterns to guide mechanistic stratification and treatment planning

**DOI:** 10.3389/fmed.2026.1898440

**Published:** 2026-09-16

**Authors:** Robert Groysman

**Affiliations:** Independent Researcher, Plano, TX, United States

**Keywords:** Long COVID, trigger-timing framework, dysautonomia, endothelial dysfunction, gut dysbiosis, mast cell activation, mechanistic stratification, mitochondrial dysfunction

## Abstract

Long COVID is characterized by substantial clinical heterogeneity, with similar symptoms often arising from different underlying physiological mechanisms. Fatigue, cognitive dysfunction, palpitations, dyspnea, gastrointestinal symptoms, sleep disruption, and exertional intolerance may each reflect autonomic dysfunction, mitochondrial or bioenergetic impairment, endothelial or microvascular dysfunction, mast cell or histamine-mediated signaling, gut dysbiosis, neuroendocrine dysregulation, or interactions among these domains. Symptom-based classification alone may therefore be insufficient for identifying dominant mechanisms or guiding treatment selection. This manuscript proposes the trigger-timing framework as a clinically accessible, hypothesis-generating method for translating patient-reported symptom behavior into mechanism-informed interpretation. The framework is based on the premise that common daily stressors, including upright posture, temperature exposure, physical or cognitive exertion, meals, emotional stress, sleep disruption, alcohol exposure, and intercurrent illness, function as real-world physiologic probes. The timing of symptom worsening after these stressors provides additional information because immediate, short-delay, same-day, delayed, cumulative, and circadian responses may reflect different response kinetics. The framework integrates four core clinical dimensions: the trigger that provokes symptom worsening, the timing of symptom onset, the symptom pattern that emerges, and the relief or recovery pattern that follows. Immediate worsening with standing, heat exposure, meals, or upright exertion, especially when relieved by lying down, supports an autonomic or circulatory pattern. Delayed worsening after physical or cognitive exertion supports impaired recovery physiology and post-exertional malaise. Meal-related symptoms may suggest autonomic, metabolic, gastrointestinal, or mast cell-related physiology depending on timing and accompanying symptoms. Circadian or persistent patterns may suggest neuroendocrine, sleep-related, autonomic, or inflammatory rhythm instability. This framework is intended as a companion operational framework to a mechanism-anchored network model of Long COVID. It may help guide targeted diagnostic evaluation, treatment prioritization, sequencing decisions, longitudinal tracking, and research cohort enrichment. The model remains conceptual and requires prospective validation to determine whether trigger-timing patterns correlate with objective physiological measures, predict differential treatment response, and improve outcomes compared with symptom-based stratification alone.

## Introduction

The companion network-disorder paper argued that symptom similarity in Long COVID does not necessarily indicate shared upstream biology and that stratification may need to be anchored in measurable physiological domains (1, 2, 29). The present manuscript uses that premise as its starting point rather than rearguing the biological network model.

The practical clinical problem addressed here is how to infer which domain may be active in an individual patient when biomarkers are incomplete, unavailable, or inconsistently standardized. A symptom such as fatigue, brain fog, palpitations, dyspnea, insomnia, gastrointestinal distress, or exertional intolerance may arise from different upstream processes and therefore requires contextual interpretation.

The trigger-timing framework proposes that symptom behavior over time provides that context. Patients with Long COVID commonly report symptoms that fluctuate with posture, exertion, meals, sleep disruption, stressors, and intercurrent illness (1, 2, 21, 22). In this framework, these daily exposures are interpreted as real-world physiologic probes because they stress autonomic, vascular, metabolic, immune, gastrointestinal, mast cell, and neuroendocrine systems.

Timing provides a second layer of mechanistic information. Immediate worsening suggests rapid regulatory failure, such as autonomic instability, vascular dysregulation, or mast cell mediator release (3, 4, 13, 18). Short-delay responses may suggest postprandial, metabolic, gastrointestinal, or histamine-mediated processes (10, 12, 13). Delayed responses, particularly after physical or cognitive exertion, suggest impaired recovery physiology and post-exertional malaise (5, 11, 23, 24, 32). Cumulative worsening may indicate limited physiologic reserve, while circadian or persistent patterns may suggest autonomic, sleep-related, inflammatory, thyroid, sex-hormone, or broader neuroendocrine rhythm disruption (9, 14, 25–28).

This manuscript proposes the trigger-timing framework as a practical method for translating observable stress-response patterns into mechanism-informed clinical hypotheses. It integrates four dimensions: the trigger that provokes symptoms, the timing of symptom onset, the symptom cluster that worsens, and the relief or recovery pattern that follows.

The framework is not intended to replace objective testing, establish a definitive diagnosis, or assign patients to mutually exclusive categories. Its purpose is to help clinicians decide which mechanisms should be evaluated first and which treatment sequence is most rational to test.

The present manuscript is therefore not a restatement of the companion network model (29). It addresses the next clinical question: how can the domains defined in that model be recognized through reproducible symptom behavior in day-to-day life?

The novelty of the trigger-timing framework does not lie in identifying new Long COVID symptoms, triggers, or biological mechanisms. Its proposed contribution is the structured integration of trigger type, response latency, symptom pattern, relief or recovery behavior, reproducibility, and objective corroboration to generate mechanism-informed hypotheses that can be prospectively tested against physiological measurements, functional outcomes, and treatment response.

Although similar trigger-response patterns may occur in other post-infectious or chronic multisystem illnesses, the present framework is anchored in Long COVID findings and related physiological literature relevant to autonomic dysfunction, exertion-related impairment, endothelial and microvascular dysfunction, persistent inflammatory signaling, gut dysbiosis, mast cell-related signaling, and neuroendocrine disruption (3–18, 29). Its Long COVID specificity lies not in claiming that each mechanism is unique to Long COVID, but in applying SARS-CoV-2-associated autonomic, vascular, immune, metabolic, gastrointestinal, mast cell-related, and neuroendocrine findings to the practical problem of interpreting daily-life symptom behavior after COVID-19.

This clinical positioning is consistent with recent calls to move Long COVID care beyond broad symptom labels toward more individualized assessment and management, while recognizing that mechanistic reasoning should complement, not replace, empirical clinical evidence and objective measurement (30, 31). Recent Long COVID studies also support the clinical relevance of delayed post-exertional physiology, orthostatic intolerance, and persistent endothelial dysfunction as distinct but overlapping physiological signals that may require different evaluation pathways (32–34).

## 2 Conceptual basis of the trigger-timing framework

### 2.1 Symptom behavior as a mechanistic signal

The trigger-timing framework begins with dynamic symptom behavior rather than with domain definitions themselves. It asks how symptoms behave under physiologic stress and whether those behaviors point toward one or more domains identified in the companion model.

The clinical question is therefore four-part: what provokes worsening, how quickly symptoms appear, which symptom cluster emerges, and what relief or recovery pattern follows.

Figure 1 summarizes how trigger-timing inputs are converted into mechanism-informed interpretation and clinical prioritization.

**Figure 1:**
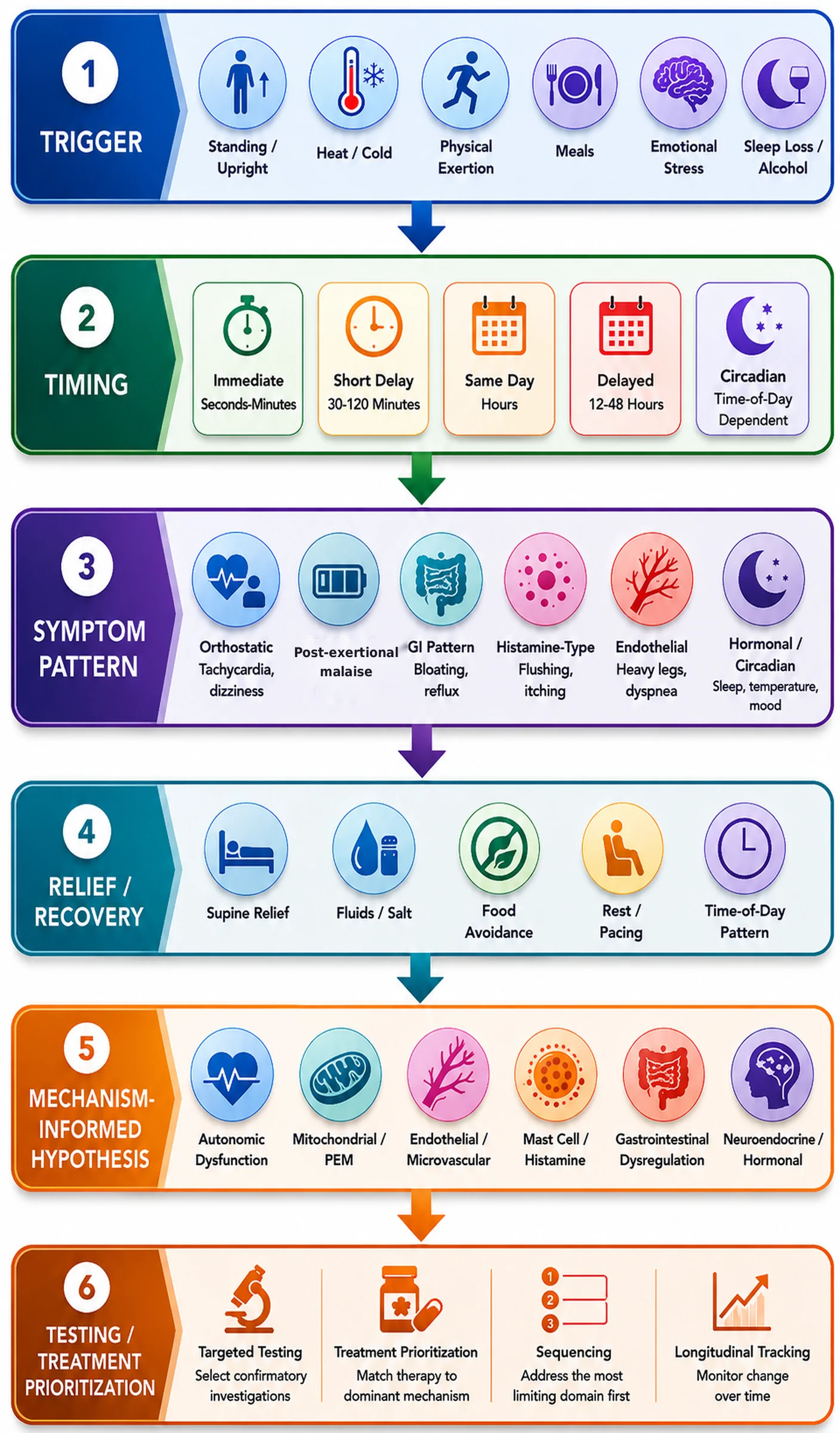
Conceptual overview of the trigger-timing framework. Physiologic triggers act as real-world stressors that challenge regulatory systems. The framework uses four core interpretive inputs: trigger, timing, symptom pattern, and relief or recovery pattern. These inputs are then translated into a mechanism-informed hypothesis and a testing or treatment-prioritization pathway. This framework is intended to support probabilistic clinical reasoning rather than establish a definitive diagnosis.

Not all symptoms in Long COVID demonstrate clear trigger dependence or temporal fluctuation. Some manifestations may remain persistent, baseline, or minimally responsive to identifiable physiologic stressors. The trigger-timing framework is therefore intended primarily for interpreting dynamic symptom behavior rather than explaining all aspects of illness expression.

The framework integrates four observable dimensions:

Trigger+Timing+SymptomPattern+ReliefPattern=Mechanism-InformedHypothesis

This equation is heuristic rather than diagnostic. It provides a structured method for converting patient-reported symptom behavior into testable clinical hypotheses. For example, fatigue that occurs immediately after standing and improves when supine suggests a different pattern than fatigue that appears 24 h after exertion and persists for several days.

### 2.2 Triggers as functional physiologic probes

The use of physiologic stressors to reveal underlying dysfunction is well established in clinical medicine. Orthostatic testing evaluates autonomic and circulatory regulation, cardiopulmonary exercise testing evaluates cardiopulmonary and metabolic responses to exertion, and glucose tolerance testing evaluates metabolic responses to carbohydrate challenge (3, 4, 9, 10, 17–19). These tests share a common principle: physiologic dysfunction may become apparent only when a regulatory system is stressed.

The trigger-timing framework extends this principle to real-world symptom behavior. Common daily triggers may function as natural, repeated physiologic probes. Upright posture challenges autonomic tone, vascular constriction, venous return, and cerebral perfusion (3, 4, 8, 18, 33). Heat exposure challenges vascular tone and thermoregulatory control. Cold exposure may activate sympathetic responses and reveal thermoregulatory or metabolic vulnerability. Physical and cognitive exertion challenge energy production, oxygen delivery, autonomic regulation, and recovery physiology (9, 17, 19, 23, 24, 32). Meals challenge glucose and insulin regulation and may also interact with gastrointestinal, immune, autonomic, and mast-cell-mediated physiology (10, 13). Emotional stress may interact with autonomic, neuroimmune, gastrointestinal-autonomic, and neuroendocrine pathways proposed as contributors to Long COVID physiology (14, 16).

Because each trigger stresses multiple systems, trigger sensitivity alone is not sufficient to identify a mechanism. Standing intolerance may reflect postural orthostatic tachycardia syndrome (POTS), hypovolemia, impaired vasoconstriction, endothelial dysfunction, or deconditioning. Meal-related symptoms may reflect splanchnic blood pooling, insulin dysregulation, histamine release, gut dysbiosis, or vagal dysfunction. Exertional intolerance may reflect dysautonomia, endothelial dysfunction, impaired oxygen extraction, mitochondrial dysfunction, or post-exertional malaise. The trigger identifies the stressor, but additional information is needed to interpret the physiologic response.

### 2.3 Timing as a marker of response kinetics

Timing provides the second major dimension of the framework. The latency between trigger exposure and symptom worsening may reflect the kinetics of the underlying physiologic process.

Immediate responses, occurring within seconds to minutes, are most consistent with rapid regulatory systems such as autonomic tone, vascular control, heart rate regulation, and mediator release (3, 4, 13, 18). For example, dizziness, tachycardia, head pressure, or brain fog that begins shortly after standing suggests orthostatic circulatory stress. Flushing, itching, tachycardia, or diarrhea that begins shortly after heat, alcohol, or food exposure may suggest mast cell or histamine-mediated physiology (13).

Short-delay responses, occurring within approximately 30–120 min, may reflect postprandial, metabolic, gastrointestinal, autonomic, or histamine-related processes (10, 13). For example, fatigue, brain fog, shakiness, tachycardia, or sleepiness after carbohydrate intake may suggest glycemic, insulin-related, autonomic, or histamine-mediated mechanisms. Bloating, reflux, abdominal pain, or diarrhea after meals may suggest gastrointestinal dysregulation, gut dysbiosis, or gut-autonomic interaction (7, 12).

Delayed responses, especially those occurring 12–48 h after physical or cognitive exertion, are particularly important because they are characteristic of post-exertional symptom exacerbation and post-exertional malaise (5, 11, 23, 24). In this pattern, the clinical clue is not only that exertion worsens symptoms, but that the worsening is delayed and often disproportionate to the activity performed. This timing pattern suggests impaired recovery physiology rather than simple exertional intolerance.

Cumulative responses occur when repeated small exposures produce progressive worsening over hours or days. This pattern may indicate limited physiologic reserve, incomplete recovery, or cross-domain amplification. Circadian responses, including predictable morning, afternoon, evening, or nighttime worsening, may suggest neuroendocrine rhythm disruption, sleep-related destabilization, autonomic imbalance, inflammatory rhythm changes, or mast cell activity (14).

### 2.4 Symptom pattern and relief pattern as mechanistic filters

The symptom pattern that emerges after a trigger further refines interpretation. The same trigger may produce different symptom clusters depending on the dominant system involved. Heat exposure followed by tachycardia, dizziness, and presyncope suggests an autonomic or circulatory pattern (3, 4, 18, 33). Heat exposure followed by flushing, itching, diarrhea, and congestion suggests a mast cell or histamine-mediated pattern (13). Heat exposure followed by heavy legs, exertional dyspnea, and head pressure may suggest vascular or microvascular involvement (34).

Relief patterns provide an additional filter. Symptoms that improve rapidly when supine support an orthostatic or circulatory mechanism, and improvement with fluids, salt, compression, or volume expansion may support hypovolemia or autonomic intolerance (3, 4, 18, 33). Symptoms that improve with fasting or specific food avoidance may suggest postprandial, gut-mediated, or histamine-related physiology (7, 10, 12, 13). Symptoms that require prolonged rest after exertion, especially when worsening occurs the next day, support impaired recovery physiology and post-exertional malaise (5, 11, 23, 24, 32).

Relief patterns are not diagnostic by themselves, but they help determine whether the suspected mechanism behaves consistently across repeated exposures. A pattern becomes more clinically meaningful when the same trigger, timing, symptom cluster, and relief pattern recur across multiple episodes.

### 2.5 Probabilistic interpretation and clinical use

Interpretation depends on convergence rather than on a single symptom or trigger. A mechanism-informed hypothesis becomes stronger when trigger, timing, symptom cluster, relief or recovery behavior, reproducibility, and objective testing point in the same direction.

Discriminative utility arises from the combination of trigger, latency, symptom cluster, and recovery behavior rather than from any single symptom or trigger. For example, heat exposure followed immediately by presyncope and supine relief suggests a different working hypothesis than heat exposure followed by flushing, itching, congestion, and diarrhea. Similarly, exertional intolerance during upright activity should be distinguished from a delayed next-day crash with prolonged recovery.

For example, immediate worsening with standing, heat, meals, and emotional stress, especially when associated with tachycardia, dizziness, upright brain fog, and relief when supine, supports an autonomic or orthostatic intolerance pattern (3, 4, 18, 33). Delayed worsening after physical or cognitive exertion, especially when associated with heavy limbs, flu-like malaise, sore throat, cognitive crash, and prolonged recovery, supports a post-exertional malaise or impaired bioenergetic recovery pattern (5, 11, 23, 24, 32). Meal-related symptoms with short-delay onset may suggest gut, metabolic, autonomic, or mast cell involvement, depending on whether the dominant symptom cluster is bloating and bowel change, glycemic-type fatigue and shakiness, tachycardia and pooling, or flushing and allergic-type symptoms (7, 10, 12, 13).

These patterns modify probability; they do not prove causality. Objective testing remains necessary when available, especially for orthostatic intolerance, cardiopulmonary limitation, endocrine abnormalities, endothelial dysfunction, mast cell activation, and gastrointestinal disease.

In this way, the framework operationalizes the companion network model by linking patient-reported stress-response behavior to mechanism-informed clinical decision-making (29).

Representative patterns are summarized in Table 1. These patterns are intended to illustrate how trigger type, timing, symptom expression, and relief or recovery behavior can be interpreted together. They should not be used as deterministic diagnostic criteria, but as structured clinical signals that increase or decrease the likelihood of specific physiological domains.

**Table 1:** Domain-level trigger-timing patterns and mechanistic associations.

Mechanism-informed pattern	Common trigger pattern	Typical timing	Symptom pattern	Relief or recovery clue
Orthostatic intolerance/POTS	Standing, heat, meals, immediate exertion	Immediate, seconds to minutes	Tachycardia, dizziness, presyncope, upright brain fog, coat-hanger pain	Improves when supine; may improve with fluids, salt, compression
Fight-or-flight/sympathetic dominance	Emotional stress, cold exposure, nighttime, exertion	Immediate or circadian	Palpitations, tremor, internal vibrations, anxiety-like activation, insomnia	Improves with down-regulation, reduced stimulation, autonomic calming
Mitochondrial dysfunction/PEM	Physical exertion, cognitive exertion, sleep deprivation	Delayed, often 12–48 h	Delayed fatigue, heavy limbs, flu-like malaise, sore throat, cognitive crash	Requires prolonged rest; worsens with repeated exertion
Endothelial/microvascular dysfunction	Heat, standing, exertion	Immediate, progressive, or same day	Heavy legs, exertional dyspnea, head pressure, poor stamina	May worsen with heat and upright activity; may overlap with autonomic symptoms
Gut dysbiosis/gut-autonomic interaction	Meals, fermentable foods, carbohydrates, stress	Short delay to delayed	Bloating, gas, reflux, abdominal pain, diarrhea or constipation, fatigue	Improves with fasting or trigger-food avoidance
MCAS/histamine signaling	Heat, meals, alcohol, stress, chemicals	Immediate to short delay	Flushing, itching, hives, congestion, tachycardia, diarrhea, headaches	Improves with trigger avoidance or antihistamine-type response
Neuroendocrine/hormonal dysregulation	Time of day, cold exposure, exertion, sleep disruption	Circadian, persistent, or cumulative	Cold intolerance, hot flashes, poor recovery, sleep disruption, mood instability, low energy	Pattern follows daily rhythm, menstrual rhythm, or persistent metabolic slowing

MCAS, mast cell activation syndrome; PEM, post-exertional malaise; POTS, postural orthostatic tachycardia syndrome.

This table illustrates representative patterns linking common physiologic triggers, timing of symptom onset, symptom pattern, and relief or recovery behavior to mechanism-informed physiologic domains. Immediate responses are generally associated with rapid regulatory processes such as autonomic or circulatory dysfunction, whereas delayed responses are more consistent with impaired recovery physiology, post-exertional malaise, or metabolic dysfunction. These associations indicate patterns that increase the likelihood of specific physiologic mechanisms and are intended to guide interpretation rather than establish a definitive diagnosis.

## 3 Expanded trigger-timing assessment for mechanism identification

### 3.1 Relationship to the prototype screening instrument

A simplified prototype mechanism-oriented screening instrument was introduced previously in the companion network model as a hypothesis-generating tool for organizing symptom-trigger patterns into mechanistically relevant domains (29). That prototype used a 0–3 rating scale and grouped items into domains including orthostatic dysautonomia, sympathetic dominance, bioenergetic dysfunction with and without post-exertional malaise, gut dysbiosis or barrier dysfunction, mast cell or histamine-mediated patterns, and neuroendocrine dysregulation.

The present manuscript expands that prototype into a more detailed operational framework by incorporating timing, symptom pattern, relief or recovery behavior, and cross-domain interpretation. This expanded framework is intended to address a key limitation of simple symptom-trigger screening: the same trigger may reflect different mechanisms depending on when symptoms appear, which symptoms worsen, and what improves them.

For example, meal-related worsening may reflect postprandial autonomic stress, glucose or insulin dysregulation, histamine-mediated signaling, gut dysbiosis, vagal dysfunction, or inflammatory activation. Exertion-related worsening may reflect immediate autonomic or circulatory limitation, endothelial or microvascular dysfunction, non-PEM bioenergetic limitation, or delayed post-exertional malaise. The expanded framework therefore requires interpretation across multiple dimensions rather than relying on isolated questionnaire items.

### 3.2 Assessment dimensions

The expanded trigger-timing assessment includes six interpretive dimensions:

Trigger exposure
Timing of symptom onset
Symptom pattern
Relief or recovery pattern
Reproducibility across episodes
Objective testing pathway

Each dimension contributes to mechanism-informed interpretation.

The first four dimensions generate the working hypothesis; reproducibility and objective testing determine how much confidence should be assigned to it.

### 3.3 Severity rating and timing categories

For each trigger domain, patients may rate the severity of symptom worsening using a semi-quantitative scale:

0 = No worsening
1 = Mild worsening
2 = Moderate worsening
  3 = Severe worsening

This scale is intended to estimate the intensity and consistency of symptom provocation. It should not be interpreted as a validated diagnostic score.

For each trigger, patients should also identify the most typical timing category:

Immediate = seconds to minutes
Short delay = 30–120 min
Same day = several hours
Delayed = 12–48 h
Cumulative = progressive worsening after repeated exposure
Circadian = predictable time-of-day pattern

A timing category should be selected only when it is reproducible across multiple episodes. If timing varies substantially, the pattern should be recorded as mixed or indeterminate. These timing categories are intended as pragmatic clinical bins rather than validated biological thresholds. They were selected to distinguish rapid regulatory responses, postprandial or short-delay responses, same-day cumulative responses, delayed post-exertional worsening, and time-of-day-dependent patterns. Future validation studies should determine whether these categories require refinement.

Importantly, the trigger-timing framework is not intended to reduce mechanistic interpretation to a rigid numerical scoring system. Trigger behavior is often contextual, overlapping, and temporally dynamic. In clinical practice, mechanistic interpretation frequently emerges from longitudinal symptom narratives, reproducible story fragments, and pattern recognition across multiple physiologic stressors rather than from isolated questionnaire responses alone. The framework is therefore intended to structure clinical reasoning rather than replace individualized history-taking or objective physiologic assessment.

### 3.4 Core trigger domains

The expanded assessment should include the following trigger domains:

Standing or upright posture
Heat exposure
Cold exposure
Physical exertion
Cognitive exertion
Meals in general
Carbohydrate-heavy meals
Fermentable or high-fiber foods
Alcohol
Emotional stress or excitement
Sleep deprivation
Nighttime or lying down
Dehydration or fluid loss
Intercurrent illness or infection
Menstrual cycle or hormonal shifts when applicable

These triggers were selected because they impose predictable physiologic demands across autonomic, vascular, metabolic, immune, gastrointestinal, mast cell, sleep, and neuroendocrine systems.

### 3.5 Operational interpretation domains inherited from the network model

The expanded assessment uses the same domain labels proposed in the companion network paper, but it does not redefine or reargue their biological evidence. They function here as clinical interpretation bins for trigger-timing patterns:

Autonomic dysfunction, including POTS, orthostatic intolerance, hypovolemia, sympathetic overactivation, and vagal dysfunction
Mitochondrial or bioenergetic dysfunction, including PEM-associated and non-PEM patterns
Endothelial or microvascular dysfunction, including impaired vascular regulation, tissue perfusion limitation, and immunothrombotic patterns (6, 20, 34)
Gut dysbiosis and barrier dysfunction, including gut-immune and gut-autonomic interaction
Mast cell activation and histamine-mediated signaling
Neuroendocrine and hormonal dysregulation, including thyroid, sex hormone, stress-axis, and circadian rhythm patterns

Secondary amplifiers are included only when they help explain broadening sensitivity, sustained flares, or cross-domain coupling; they should not automatically override the dominant trigger-timing domain.

### 3.6 Interpretation rules

The representative table should be interpreted using several rules.

First, trigger-timing patterns are probabilistic. A pattern increases the likelihood of a mechanism but does not prove it.

Discordant, inconsistent, or poorly reproducible trigger-timing observations should lower confidence in mechanism assignment rather than be forced into a predefined category. Such patterns should be recorded as mixed or indeterminate and should prompt broader differential diagnosis and greater reliance on objective corroboration. The framework is intended to constrain, rather than encourage, arbitrary interpretation of isolated patient-reported symptoms.

Second, mechanisms are not mutually exclusive. A patient may show strong autonomic, mitochondrial, gut, and mast cell patterns at the same time.

In practical use, domain dominance is inferred from convergence among reproducibility, timing specificity, symptom-pattern specificity, functional impact, relief or recovery behavior, and objective corroboration when available, rather than from symptom count alone.

Third, timing often provides the strongest discriminator. Immediate exertional symptoms and delayed exertional crashes should not be treated as the same pattern.

Fourth, relief and recovery patterns help refine the hypothesis. Supine relief strengthens an orthostatic interpretation. Food avoidance strengthens a gut or histamine interpretation. Prolonged rest after delayed exertional worsening strengthens a PEM interpretation.

Fifth, secondary amplifiers should be considered when symptoms become more widespread, more easily triggered, or less predictable. Sleep disruption, immune activation, neuroinflammation, small fiber neuropathy, and inflammatory flares may lower the threshold for multiple domains to react.

Sixth, the table should guide testing and treatment prioritization, not replace clinical judgment.

### 3.7 Representative bedside trigger-timing patterns

A shortened representative version of the trigger-timing framework is shown below for readability within the main manuscript. An expanded operational version containing additional trigger combinations, timing behaviors, symptom patterns, and mechanism-informed interpretations is provided as Supplementary Table 1. The patterns shown in Table 2 are intentionally representative rather than exhaustive. Individual patients may demonstrate overlapping, evolving, or mixed trigger-timing behaviors involving multiple physiological domains simultaneously.

**Table 2:** Representative bedside trigger-timing patterns in long COVID.

Trigger	Timing pattern	Representative symptom pattern	Relief/recovery clue	Mechanism-informed interpretation
Standing/upright posture	Immediate	Tachycardia, dizziness, upright brain fog, presyncope	Improves when supine	Orthostatic intolerance/POTS pattern
Standing/upright posture	Cumulative or same day	Progressive fatigue, heavy legs, exertional dyspnea	Improves with fluids, salt, compression, lying down	Autonomic plus endothelial/circulatory overlap
Heat exposure	Immediate	Tachycardia, weakness, presyncope	Improves with cooling or lying down	Autonomic/circulatory instability
Heat exposure	Immediate to short delay	Flushing, itching, congestion, diarrhea	Improves with trigger avoidance or antihistamine-type response	Mast cell/histamine-mediated pattern
Physical exertion	Immediate	Tachycardia, dyspnea, head pressure	Improves with stopping exertion	Autonomic, circulatory, or perfusion limitation
Physical exertion	Delayed 12–48 h	Heavy limbs, cognitive crash, flu-like malaise, worsened pain	Requires prolonged recovery	PEM/impaired bioenergetic recovery
Cognitive exertion	Immediate or delayed	Overstimulation, head pressure, cognitive crash	Improves with cognitive rest	Sympathetic activation or PEM-type cognitive intolerance
Meals in general	Immediate or short delay	Tachycardia, fatigue, shakiness, sleepiness	Improves with meal modification or smaller meals	Postprandial autonomic or metabolic pattern
Meals/alcohol/heat	Immediate to short delay	Flushing, itching, congestion, diarrhea, tachycardia	Improves with avoidance or antihistamine-type response	Mast cell/histamine-mediated pattern
Fermentable or high-fiber foods	Delayed	Bloating, reflux, abdominal pain, bowel changes	Improves with trigger-food avoidance	Gut dysbiosis/gut-autonomic interaction
Emotional stress	Immediate	Palpitations, tremor, insomnia, internal vibrations	Improves with autonomic down-regulation	Sympathetic dominance/fight-or-flight pattern
Emotional stress	Delayed or same day	Fatigue, inflammatory-type flare, brain fog	Requires recovery time	Neuroimmune or stress-axis amplification
Sleep deprivation	Next day	Brain fog, orthostatic worsening, fatigue	Improves with sleep stabilization	Sleep-related cross-domain amplification
Nighttime/circadian pattern	Circadian	Hyperarousal, insomnia, temperature instability	Follows time-of-day pattern	Circadian or neuroendocrine dysregulation
Menstrual or hormonal shifts	Cyclic	Migraine, fatigue, mood change, heat intolerance	Predictable cycle relationship	Hormonal/neuroendocrine modulation

PEM, post-exertional malaise; POTS, postural orthostatic tachycardia syndrome.

This table provides representative bedside examples of how trigger exposure, timing pattern, symptom expression, and relief or recovery behavior may be integrated to generate mechanism-informed clinical interpretations. The patterns are illustrative rather than exhaustive and should be interpreted probabilistically, especially when multiple physiological domains overlap in the same patient.

Tables 1, 2 serve complementary purposes. Table 1 is organized from the physiological-domain perspective and summarizes the trigger-timing characteristics associated with each mechanism-informed domain. Table 2 reverses the direction of reasoning and begins with the bedside observation, particularly the trigger and temporal response, before moving toward a mechanism-informed interpretation. Some examples therefore appear in both tables because they illustrate the same relationship from opposite clinical starting points.

### 3.8 Pattern-weighted interpretation

Domain-level interpretation can incorporate the severity ratings of items that map to each mechanism, but numerical totals should not be treated as sufficient by themselves. Interpretation should be weighted by timing specificity, symptom pattern, relief behavior, reproducibility, and clinical context.

High-specificity patterns should carry greater interpretive weight when a characteristic trigger, latency, symptom pattern, and relief or recovery behavior converge. For example, immediate standing symptoms relieved by lying down provide a stronger autonomic signal than fatigue alone, whereas a delayed exertional crash with prolonged recovery provides a stronger PEM or bioenergetic signal than immediate exertional fatigue. Additional domain-specific examples are summarized in Tables 1, 2.

Lower-specificity symptoms such as fatigue, brain fog, insomnia, or generalized pain should not drive domain assignment unless they occur in a reproducible trigger-timing pattern.

The proposed pattern-weighted approach is illustrative and should not be interpreted as a validated quantitative instrument. At this stage, ratings are intended to support structured clinical reasoning and hypothesis generation. Prospective studies will be required to determine item weighting, inter-rater reliability, test-retest reliability, and correlation with objective physiological measures.

### 3.9 Clinical output of the expanded assessment

The output of the expanded assessment should include:

Dominant mechanism-informed pattern
Secondary contributing patterns
Possible cross-domain amplifiers
Recommended confirmatory testing
Initial treatment-prioritization logic
Tracking markers for follow-up

Once a dominant pattern is identified, the output should specify the most appropriate confirmatory testing, initial treatment-prioritization logic, and longitudinal tracking markers. Detailed examples of pattern-to-testing and treatment pathways are provided in Tables 2, 3.

**Table 3:** Mechanism-informed testing and treatment prioritization pathways.

Dominant pattern	Testing priority	Treatment planning emphasis	Tracking marker
Autonomic/orthostatic intolerance	Active stand, tilt-table testing, heart rate and blood pressure monitoring, autonomic testing (3, 4, 18, 33)	Volume support, orthostatic countermeasures, autonomic stabilization, avoidance of excessive upright stress during early treatment	Standing tolerance, heart rate response, upright brain fog, presyncope frequency
Sympathetic dominance/fight-or-flight pattern	Heart rate variability, catecholamines in selected cases, sleep and stress pattern review (3, 4, 14, 18)	Down-regulation of sympathetic activation, sleep stabilization, avoidance of excessive stimulation, treatment of overlapping triggers	Internal tremor, palpitations, nighttime hyperarousal, stress-triggered flares
Post-exertional malaise/impaired recovery physiology	Clinical PEM assessment, functional capacity assessment, CPET when clinically appropriate and safe (5, 9, 11, 17, 19, 23, 24, 32)	Pacing, crash prevention, avoidance of aggressive graded escalation, support of recovery physiology	Crash frequency, crash latency, recovery duration, functional baseline stability
Endothelial/microvascular pattern	Vascular or endothelial assessment where available, perfusion-related evaluation, exertional limitation assessment (6, 20, 34)	Vascular and perfusion-directed treatment strategy, reduction of heat and upright stress, attention to overlap with autonomic and mitochondrial domains	Heavy legs, exertional dyspnea, head pressure, heat intolerance, stamina
Gut dysbiosis/gut-autonomic pattern	Gastrointestinal evaluation, stool or microbiome testing where appropriate, breath testing when indicated, dietary trigger review (7, 10, 12, 13)	Gut-directed strategy, food trigger identification, motility support, barrier and microbiome-focused interventions	Bloating, reflux, bowel pattern, meal-related fatigue, delayed food reactions
Mast cell/histamine pattern	Mediator testing when feasible, symptom-timed evaluation, exposure and food trigger review (8, 9, 13)	Trigger reduction, histamine- or mast-cell-directed strategy when clinically appropriate, attention to alcohol and heat sensitivity	Flushing, itching, congestion, diarrhea, tachycardia, multisystem flares
Neuroendocrine/hormonal pattern	Thyroid testing, cortisol rhythm assessment where appropriate, sex hormone evaluation, sleep and circadian assessment (9, 14, 25–28)	Correction of objective endocrine abnormalities, sleep and rhythm stabilization, attention to cycle-related or time-of-day patterns	Cold intolerance, heat intolerance, sleep quality, libido, menstrual pattern, morning or nighttime worsening

CPET, cardiopulmonary exercise testing; HRV, heart rate variability; PEM, post-exertional malaise.

This table illustrates how dominant trigger-timing patterns may guide testing priorities, treatment-planning emphasis, and longitudinal tracking markers. Testing examples reflect objective assessment pathways discussed in the text and are cited in the Testing Priority column. The examples are intended to support prioritization logic rather than prescribe standardized diagnostic or treatment protocols. Specific interventions should be individualized, clinically supervised, and guided by confirmatory testing, contraindications, comorbidities, illness severity, patient tolerance, and evolving clinical response.

The purpose of this output is not to label the patient with a single disease category. The purpose is to identify which mechanism is most likely driving symptom behavior and which treatment sequence is most rational to test first.

### 3.10 Practical bedside use of the trigger-timing framework

In clinical practice, the trigger-timing framework can be applied as a structured history-taking sequence rather than as a stand-alone score. The clinician first identifies the most reproducible triggers, then assigns the usual timing pattern, defines the symptom cluster that emerges, and asks what reliably improves or shortens the episode. These elements are then integrated to identify the most likely dominant mechanism-informed pattern and the most important overlapping contributors.

A practical sequence is: identify the recurring trigger; determine whether onset is immediate, short-delay, same-day, delayed, cumulative, or circadian; match the symptom cluster to a plausible physiological domain; assess relief or recovery behavior; decide which mechanism appears most reproducible and clinically limiting; choose confirmatory testing; select the first treatment direction; and track whether the same trigger produces a less severe, shorter, or more delayed response over time.

This bedside use is especially important when biomarkers are unavailable, incomplete, or inconsistently standardized. It allows clinicians to convert patient narratives into testable hypotheses while preserving the need for objective assessment, clinical judgment, and longitudinal reassessment.

## 4 Translating trigger-timing patterns into testing and treatment prioritization

### 4.1 Rationale for mechanism-guided clinical action

The purpose of the trigger-timing framework is not only to classify symptom behavior, but to organize clinical decision-making around the most likely physiological drivers of symptom worsening. In heterogeneous conditions such as Long COVID, treatment selection based on symptoms alone may be imprecise because similar symptoms can arise from different mechanisms. Fatigue, cognitive dysfunction, palpitations, dyspnea, insomnia, and gastrointestinal symptoms may each reflect autonomic, metabolic, vascular, immune, gastrointestinal, mast cell, endocrine, or sleep-related processes (1, 2, 14–16, 21, 22, 30).

A mechanism-informed approach begins by identifying the dominant trigger-timing pattern and then selecting confirmatory testing or therapeutic priorities based on that pattern. This does not imply that the trigger-timing assessment establishes a definitive diagnosis. Rather, it functions as a structured clinical reasoning tool that helps determine which mechanism should be evaluated first and which treatment pathway is most rational to prioritize.

This is especially important because many patients demonstrate overlapping mechanisms. In such cases, attempting to treat every possible domain simultaneously may obscure treatment response, worsen tolerability, or make it difficult to determine which intervention produced benefit or harm. The trigger-timing framework therefore supports treatment sequencing, with initial attention directed toward the mechanism that appears most reproducible, most limiting, or most likely to destabilize other domains.

### 4.2 From pattern recognition to confirmatory testing

Once a reproducible trigger-timing phenotype has been identified using the preceding framework, the next step is to determine which objective evaluation would most directly confirm, refine, or challenge the working hypothesis. Trigger-timing patterns should guide, rather than replace, objective evaluation.

For autonomic or orthostatic patterns, testing may include active stand testing, tilt-table testing, heart rate and blood pressure monitoring, heart rate variability assessment, and autonomic reflex testing where available (3, 4, 18, 33).

For exertional intolerance and suspected impaired recovery physiology, evaluation may include cardiopulmonary exercise testing, functional capacity assessment, exertional lactate dynamics, and careful clinical assessment for post-exertional symptom exacerbation (9, 17, 19, 23, 24, 32).

For meal-related patterns, testing may include postprandial heart rate and blood pressure monitoring, metabolic or glycemic assessment when indicated, and gastrointestinal evaluation guided by the associated symptom pattern (7, 10, 12).

For mast cell or histamine-type patterns, clinical evaluation may consider symptom-timed mediator testing when feasible, together with structured food and exposure tracking (13).

For neuroendocrine or hormonal patterns, testing may include thyroid evaluation, cortisol rhythm assessment when clinically appropriate, sex hormone assessment, and evaluation of sleep and circadian stability (14, 25–28).

### 4.3 Treatment prioritization principles

Treatment planning within this framework should follow several principles.

First, treatment should be prioritized according to the dominant reproducible pattern rather than the longest symptom list. The purpose is to select a rational first treatment direction while preserving attention to secondary and overlapping domains.

Second, timing should influence sequencing. Immediate autonomic or circulatory patterns and delayed impaired-recovery patterns may require different evaluation and management priorities (5, 11, 23, 24).

Third, treatment should account for cross-domain interactions without assuming that every domain must be treated at once. The operational question is which active pattern must be stabilized first to make the next intervention tolerable and interpretable.

Fourth, treatment response should be tracked using the same trigger-timing variables used to define the baseline pattern, including trigger tolerance, symptom latency, severity, recovery duration, and functional consequences.

### 4.4 Mechanism-informed treatment pathways

The framework does not prescribe a single standardized treatment protocol or imply that trigger-timing patterns alone establish a definitive mechanism. Instead, it organizes treatment planning around the most reproducible and physiologically plausible pattern of symptom behavior. The pathways shown in Table 3 are intended to illustrate mechanism-informed prioritization logic rather than fixed therapeutic algorithms. Treatment selection should remain individualized and guided by objective testing where available, comorbidities, contraindications, illness severity, treatment tolerance, and evolving clinical response.

### 4.5 Sequencing in mixed mechanism presentations

Many patients will not fit a single domain. Mixed patterns are expected and should not be treated as a failure of the framework. Instead, the goal is to identify which mechanism appears most upstream, most destabilizing, or most limiting at the current stage of illness.

When autonomic instability is severe, it may need to be addressed early because it can interfere with activity tolerance, sleep, gastrointestinal function, and cognitive function. When PEM is prominent, treatment sequencing should avoid interventions that increase exertional demand before the patient's recovery physiology is stabilized. When mast cell or histamine symptoms are prominent, tolerability may improve only after exposure-related flares are reduced. When gastrointestinal symptoms dominate, meal-related triggers may need to be addressed before more aggressive metabolic or supplement strategies are tolerated.

In this approach, the first treatment target is not necessarily the mechanism with the highest theoretical importance. It is the mechanism that most clearly drives reproducible symptom worsening and limits the patient's ability to tolerate further evaluation or treatment.

### 4.6 Longitudinal use of trigger-timing patterns

The trigger-timing framework can also be used longitudinally. At baseline, the framework helps identify the dominant mechanism-informed pattern. During treatment, it helps determine whether the pattern is changing.

A meaningful response may appear as reduced severity after the same trigger, longer latency before symptom onset, shorter recovery time, reduced cross-domain spread, or increased tolerance before a crash occurs. For example, improvement in orthostatic physiology may appear as longer standing tolerance or reduced postprandial tachycardia. Improvement in PEM physiology may appear as fewer next-day crashes, reduced crash duration, or improved baseline stability. Improvement in histamine or gut physiology may appear as fewer food-triggered multisystem flares.

Conversely, worsening of trigger sensitivity may indicate that the current treatment is poorly tolerated, that a secondary amplifier has become more active, or that the patient's physiologic reserve has declined. In this way, trigger-timing patterns can serve as practical clinical tracking markers even when objective biomarkers are unavailable or inconsistent.

### 4.7 Clinical output

The clinical output of the trigger-timing assessment should identify the dominant mechanism-informed pattern, secondary contributing patterns, possible cross-domain amplifiers, recommended confirmatory testing, initial treatment-prioritization logic, and tracking markers for follow-up.

These outputs should be interpreted as mechanism-informed clinical guidance rather than diagnostic labels. The framework is intended to support individualized treatment planning, research enrichment, and prospective validation. Its clinical utility will depend on whether trigger-timing patterns correlate with objective physiological measures and whether mechanism-guided treatment prioritization improves outcomes compared with symptom-based management alone.

## 5 Research applications and prospective validation

### 5.1 Role in mechanism-informed cohort enrichment

The companion network paper proposed biological stratification and trial enrichment at the domain level. The present paper adds a practical entry point for that process: reproducible trigger-timing behavior.

A trigger-timing approach may allow investigators to enrich cohorts based on functional patterns rather than symptoms alone. For example, patients with delayed post-exertional worsening could be analyzed separately from patients with immediate exertional tachycardia or dyspnea. These two groups may both report exertional intolerance, but the timing pattern suggests different physiology. Immediate exertional symptoms may reflect autonomic, circulatory, cardiopulmonary, or vascular limitation, whereas delayed worsening suggests impaired recovery physiology and post-exertional malaise (5, 9, 11, 17, 19, 23, 24, 32).

Similarly, patients with meal-related symptoms could be stratified based on whether the dominant pattern is immediate tachycardia, short-delay carbohydrate-related fatigue, histamine-type flushing or diarrhea, or delayed bloating and gastrointestinal symptoms. Each pattern may suggest a different biological pathway and therefore a different therapeutic target (7, 10, 12, 13).

### 5.2 Improving trial design and treatment signal detection

Mechanism-informed stratification may improve clinical trial design by aligning study entry criteria with the mechanism targeted by the intervention. Trials of autonomic therapies may be more interpretable if participants demonstrate reproducible immediate orthostatic, heat, or postprandial autonomic patterns. Trials of mitochondrial or bioenergetic interventions may be more informative if participants demonstrate delayed post-exertional symptom exacerbation rather than nonspecific fatigue alone. Trials of gut-directed or mast cell-directed therapies may be more interpretable if participants demonstrate consistent meal-related or exposure-related trigger patterns.

This approach does not require that trigger-timing patterns replace objective biomarkers. Instead, they may be used as an enrichment layer before or alongside objective testing. A participant could be selected because the clinical pattern suggests a domain, and then the domain could be evaluated using physiological testing, laboratory markers, or functional measures where available.

The framework may therefore support a two-step research strategy:

Identify candidate mechanism-enriched groups using trigger-timing patterns
Validate those groups using objective physiological or biomarker measures

This strategy may be especially useful in Long COVID because biomarkers are heterogeneous, may not be uniformly available, and may not correlate consistently with symptom severity across all patients (14–16).

### 5.3 Candidate validation questions, objective measures, and study approaches

Prospective validation should test whether trigger-timing patterns correlate with objective physiological measures and clinical outcomes. Different domains would require different validation endpoints.

Validation may occur at multiple biological and clinical levels. Molecular biomarkers represent one potential validation layer, but construct and predictive validity can also be examined using physiological measurements, functional endpoints, wearable data, reproducibility, treatment-response differences, and longitudinal outcomes. A clinical stratification framework need not uniquely identify a molecular pathway in order to generate empirically testable predictions.

Candidate validation pathways are summarized in Table 4.

**Table 4:** Candidate validation questions, objective measures, and study approaches.

Validation question or pattern	Candidate objective measures	Example study approach and outcomes
Immediate standing, heat worsening, or supine-relieved orthostatic symptoms	Active stand test, tilt-table testing, heart rate and blood pressure response, heart rate variability, autonomic reflex testing	Compare trigger-timing profiles with active stand or tilt-table results; track standing tolerance, presyncope frequency, upright brain fog, and postural symptom burden.
Delayed post-exertional worsening	Functional capacity assessment, CPET when appropriate, exertional lactate dynamics, activity tracking, PEM questionnaires	Compare PEM-pattern groups with functional capacity measures, CPET where appropriate, or activity tracking; track crash frequency, crash severity, recovery duration, and functional baseline stability.
Meal-related timing patterns	Postprandial heart rate and blood pressure monitoring, glucose or insulin assessment when indicated, gastrointestinal evaluation, breath testing when indicated, stool or microbiome testing where appropriate	Combine meal-trigger logs with postprandial vitals, glucose assessment, gastrointestinal evaluation, and mediator testing to distinguish autonomic, glycemic, gut, and histamine-related subgroups; track meal tolerance and delayed food-related flares.
Histamine-type trigger pattern	Symptom-timed mediator testing when feasible, food and exposure tracking	Evaluate whether classic heat, alcohol, food, flushing, itching, congestion, diarrhea, headache, or tachycardia patterns predict mediator findings or differential response to histamine-directed treatment.
Endothelial or microvascular pattern	Endothelial function testing, microvascular perfusion assessment where available, exertional limitation measures	Test whether heavy legs, exertional dyspnea, head pressure, heat intolerance, and poor stamina correlate with objective vascular or perfusion measures and vascular-directed outcomes.
Neuroendocrine or circadian pattern	Thyroid panel, sex hormone testing, cortisol rhythm assessment when appropriate, sleep assessment	Compare morning, nighttime, menstrual, temperature, sleep, and mood patterns with endocrine or sleep measures; track rhythm stability, sleep quality, and temperature intolerance.
Treatment matching and longitudinal change	Prospective symptom diaries, wearable physiological data, repeated functional measures, repeated orthostatic or activity metrics	Compare mechanism-enriched groups with symptom-defined cohorts and evaluate whether changes in trigger sensitivity, latency, recovery time, and cross-domain spread track clinical improvement or decline.

CPET, cardiopulmonary exercise testing; PEM, post-exertional malaise.

This table summarizes candidate validation pathways for testing whether reproducible trigger-timing patterns correlate with objective physiological measures, clinical outcomes, or differential treatment response. The goal of validation is not to prove that each pattern maps uniquely to one biomarker, but to determine whether trigger-timing groups show greater biological coherence or clinical utility than symptom-defined groups alone.

The key validation question is not whether every patient with a trigger-timing pattern has the same biomarker abnormality. Rather, the question is whether patients grouped by reproducible trigger-timing patterns demonstrate greater biological coherence or treatment responsiveness than patients grouped by symptoms alone.

### 5.4 Use in longitudinal monitoring

The trigger-timing framework may also serve as a longitudinal measurement tool. Many Long COVID symptoms fluctuate over time, and single time-point assessments may miss important changes in physiologic reserve. Repeated assessment of trigger sensitivity, timing, symptom pattern, and recovery behavior may provide a practical way to monitor disease trajectory.

Clinically meaningful improvement may appear as:

Reduced symptom severity after the same trigger
Longer tolerance before symptom onset
Shorter recovery time
Less delayed worsening after exertion
Fewer cumulative crashes
Reduced spread across multiple symptom domains
Improved tolerance of meals, posture, heat, exertion, or sleep disruption

Conversely, worsening may appear as lower trigger thresholds, longer recovery time, more frequent delayed crashes, more severe postprandial symptoms, increased heat intolerance, or broader multisystem flares. These changes may help clinicians and investigators determine whether a treatment is improving the dominant mechanism, worsening tolerance, or unmasking a secondary amplifier.

Longitudinal application does not require patients to identify changes in underlying pathophysiology. It records observable changes in response behavior, including trigger tolerance, latency before symptom onset, symptom severity, recovery duration, frequency of delayed worsening, and functional consequences. The validity of these measures should be evaluated against objective physiological, wearable, and functional measures rather than assumed from patient perception alone. Prospective or near-real-time symptom recording may also reduce some of the recall error associated with longer retrospective reporting periods (41).

### 5.5 Integration with biomarker and symptom-based approaches

Symptom clustering, biomarker testing, and trigger-timing assessment answer different questions: what symptoms co-occur, what biological abnormalities can be measured, and how the system responds to real-world stressors.

Integrated together, these approaches may improve stratification. For example, a patient with fatigue and brain fog could be classified descriptively by symptom cluster, functionally by trigger-timing pattern, and biologically by objective measures such as orthostatic testing, exertional testing, endocrine testing, vascular assessment, or gastrointestinal evaluation.

This layered approach may be particularly useful in a network disorder model, where multiple domains may be active at the same time and where dominant mechanisms may shift over time.

### 5.6 Hypothesis-generating predictions

The framework generates several testable predictions.

First, patients with immediate standing-related symptoms and supine relief should show higher rates of objective orthostatic abnormalities than patients selected by fatigue alone (3, 4, 18, 33).

Second, patients with delayed post-exertional worsening should differ physiologically and clinically from patients with immediate exertional intolerance without delayed recovery failure (5, 11, 23, 24, 32).

Third, meal-related symptoms should separate into distinguishable subgroups based on timing and symptom pattern, including autonomic, glycemic, gastrointestinal, and histamine-mediated patterns (7, 10, 12, 13).

Fourth, treatment response should be stronger when the therapy matches the dominant trigger-timing pattern than when the same therapy is applied to broad symptom-defined cohorts.

Fifth, changes in trigger-timing patterns over time should correlate with functional improvement or decline more closely than symptom presence alone.

These predictions are prospectively falsifiable. The framework would be unsupported if trigger-timing-defined groups do not demonstrate greater physiological or biological coherence than symptom-defined comparison groups, if predicted phenotypes cannot be distinguished using relevant objective measures, if mechanism-matched treatment does not improve response discrimination, or if longitudinal changes in trigger-timing behavior do not correspond to objective or functional change. Negative findings of this type would require modification or rejection of the relevant components of the framework.

### 5.7 Positioning as a companion operational framework

The trigger-timing framework is intended to serve as a companion operational framework to the mechanism-anchored network model of Long COVID (29). The network model identifies interacting physiological domains and secondary amplifiers. The present framework provides a method for recognizing which domains may be active in an individual patient based on observable symptom behavior.

This sister-paper relationship defines the novelty of the present manuscript. The contribution is not the identification of a new biological mechanism, but the operationalization of mechanism-informed stratification using real-world stress-response patterns. In this way, the framework can support three related goals: clinical reasoning, treatment planning, and research cohort enrichment. First, it can help identify likely dominant and overlapping mechanisms in individual patients. Second, it can help prioritize confirmatory testing, sequencing decisions, and follow-up tracking markers. Third, it can support research design by enriching cohorts according to mechanism-informed stress-response patterns and by testing whether such enriched groups demonstrate differential treatment response.

## 6 Limitations and future directions

### 6.1 Conceptual and hypothesis-generating nature of the framework

The trigger-timing framework is intended as a hypothesis-generating clinical and research model rather than a validated biological taxonomy. It does not claim that trigger-timing patterns establish definitive mechanisms, prove causality, or replace objective physiological testing. Instead, the framework proposes that reproducible patterns of symptom worsening, timing, symptom expression, and recovery behavior may provide clinically useful signals that can guide mechanistic hypotheses.

This framing is particularly important because trigger-timing categories remain working hypotheses, not mechanism labels.

### 6.2 Reliance on patient-reported symptom patterns

A major limitation of the framework is that it relies heavily on patient-reported symptom behavior. Patient-reported data are clinically valuable because the medical history remains a foundational component of clinical assessment, particularly for symptom onset, provoking factors, temporal relationships, relieving factors, episodic responses, and recovery behavior. However, trigger and timing reports may be affected by recall bias, variable symptom awareness, inconsistent exposure intensity, medication effects, activity pacing, comorbid conditions, and environmental context. Retrospective symptom ratings can differ from prospectively recorded symptom experience, especially as the recall period lengthens (41).

For example, a patient may report exertional worsening, but the exertion may vary substantially in intensity, duration, posture, temperature, emotional stress, and recovery conditions. Similarly, meal-related symptoms may vary depending on meal composition, timing, portion size, hydration status, histamine content, carbohydrate load, gastrointestinal motility, and baseline autonomic state. These variables may complicate interpretation.

Patient-reported trigger-timing information should therefore be regarded as a source of candidate physiological hypotheses rather than as an objective physiological measurement. When feasible, future validation studies should use prospective symptom diaries, standardized trigger logs, wearable physiological monitoring, activity tracking, orthostatic vital signs, or other contemporaneous measures to improve reliability. The validity of the framework depends not on assuming that patient recall is accurate, but on determining whether reproducible historical patterns correspond to objective physiological or functional findings (41).

Trigger interpretation may be particularly challenging when multiple physiologic stressors occur close together in time. Meals, exertion, emotional stress, sleep disruption, temperature exposure, and upright activity frequently overlap in daily life, and smaller stressors may cumulatively lower physiologic reserve before a larger symptomatic flare occurs. In such cases, interpretation may require longitudinal pattern recognition across repeated exposures rather than attribution to a single isolated trigger event.

### 6.3 Non-specificity of individual triggers

Individual triggers are rarely mechanism-specific. Upright posture may provoke symptoms through POTS, hypovolemia, impaired vasoconstriction, endothelial dysfunction, deconditioning, medication effects, or anemia. Meals may provoke symptoms through splanchnic pooling, glucose or insulin dysregulation, gut dysbiosis, mast cell activation, reflux, vagal dysfunction, or food intolerance. Exertion may worsen symptoms through autonomic limitation, cardiopulmonary disease, endothelial dysfunction, impaired oxygen extraction, mitochondrial dysfunction, or post-exertional malaise (3–5, 7, 9–13, 17, 19, 23, 24, 32, 34).

The framework should also be applied after appropriate evaluation for alternative or coexisting causes of symptoms, including cardiopulmonary disease, anemia, medication effects, endocrine disease, primary gastrointestinal disease, sleep disorders, and other conditions that may mimic or amplify Long COVID symptom patterns. Timing may provide important discriminatory value, especially when distinguishing immediate exertional intolerance from delayed post-exertional symptom exacerbation (5, 11, 23, 24, 32).

For this reason, the framework should not interpret trigger sensitivity alone as diagnostic. Trigger type must be interpreted together with timing, symptom pattern, relief or recovery behavior, reproducibility, and clinical context.

### 6.4 Overlapping mechanisms and cross-domain interactions

Overlap is expected because the companion model conceptualizes Long COVID as interacting domains. Trigger-timing interpretation should therefore be considered a readout of an interacting physiological network rather than a one-domain assignment. In this paper, overlap is handled operationally by identifying dominant and secondary trigger-timing patterns at the current visit rather than forcing a single category.

Domain dominance should be interpreted as a clinical working priority, not as an exclusive biological assignment. Concordant abnormalities across several domains may reflect synergistic or bidirectional coupling rather than independent mechanisms.

Dominance may also shift over time, so reassessment should focus on whether the reproducible trigger-timing pattern has changed. Comorbid conditions may alter trigger thresholds, symptom expression, response latency, or recovery behavior. Cardiopulmonary disease, anemia, medication effects, endocrine disease, primary gastrointestinal disease, sleep disorders, and other alternative or coexisting conditions must therefore remain part of the clinical interpretation.

### 6.5 Variability in objective testing

Objective testing is essential for validation, but many relevant tests have limitations. Orthostatic testing protocols vary by duration, posture, hydration status, medication use, and diagnostic thresholds (3, 4, 18, 33). Cardiopulmonary exercise testing may provide valuable information, but it may not be appropriate or safe for all patients with severe post-exertional malaise (9, 17, 19, 23, 24, 32). Mast cell mediator testing may be difficult to interpret because mediator release can be episodic and sample timing may affect results (13). Gastrointestinal and microbiome testing vary in methodology and clinical interpretability (7, 12). Endocrine testing may identify abnormalities in some patients but may not capture tissue-level signaling or dynamic hormonal or neuroendocrine rhythm disruption (14, 25–28).

These limitations do not invalidate the framework, but they emphasize the need for careful prospective validation using standardized protocols.

### 6.6 Treatment response cannot be assumed from pattern assignment

Although the framework is designed to guide treatment prioritization, pattern assignment should not be interpreted as proof that a patient will respond to a specific therapy. A patient with an autonomic-dominant pattern may not respond to a given autonomic intervention. A patient with delayed post-exertional worsening may not respond to a mitochondrial-supportive or pacing-based strategy in a predictable way. A patient with histamine-type symptoms may have overlapping mast cell, gut, autonomic, or vascular contributors.

Treatment response depends on disease stage, severity, comorbidities, medication tolerance, environmental exposures, sleep stability, immune activity, and cross-domain interactions. The framework should therefore be used to prioritize rational next steps, not to guarantee therapeutic response.

### 6.7 Need for prospective validation

Prospective validation is required to determine whether trigger-timing patterns correspond to measurable physiological domains and whether mechanism-informed treatment planning improves outcomes. Future studies should evaluate whether patients grouped by trigger-timing patterns demonstrate greater biological coherence than patients grouped by symptoms alone.

Representative validation questions, objective measures, study approaches, and outcomes are summarized in Table 4.

The framework would be strengthened if trigger-timing groups show reproducible associations with objective measures or differential treatment response. Conversely, if such patterns do not improve biological coherence or treatment prediction, the model would require revision.

### 6.8 Future development of pattern-weighted assessment

The proposed pattern-weighted assessment approach should be considered preliminary. Future work should determine how much weight should be assigned to trigger severity, timing specificity, symptom pattern, relief behavior, reproducibility, and objective test results. High-specificity patterns, such as delayed post-exertional worsening with prolonged recovery or immediate orthostatic symptoms relieved by lying down, may deserve greater weight than nonspecific symptoms such as fatigue or brain fog alone.

Future versions of the model may also incorporate wearable data, orthostatic vitals, activity measures, sleep data, heart rate variability, glucose monitoring, symptom diaries, and laboratory markers. A validated model could eventually generate domain probability estimates, identify mixed-mechanism patterns, and track response over time. Future validation should also evaluate inter-rater reliability, test-retest reliability, internal consistency where applicable, sensitivity to change, and correlation with objective physiological measures.

### 6.9 Sex, gender, and hormonal state as modifiers of trigger-timing phenotypes

Sex-related biological differences may influence the prevalence, expression, and physiological consequences of several domains incorporated into the trigger-timing framework, including autonomic dysfunction, endothelial and vascular regulation, immune signaling, and neuroendocrine function (35–38). Hormonal state may further modify temperature sensitivity, orthostatic tolerance, sleep, migraine, exertional responses, and other trigger-related symptom patterns. Menstrual or hormonal shifts are already included as potential triggers within the framework, but future validation should more explicitly record sex, gender, reproductive and menopausal status when applicable, and relevant hormonal therapies. These variables should be evaluated as potential modifiers of trigger-timing phenotypes rather than used as mechanistic assignments by themselves. Gender-related differences in symptom reporting, healthcare experiences, activity patterns, psychosocial context, and other relevant factors should also be considered when interpreting patient-reported trigger-timing information.

### 6.10 Potential implications for healthcare pathways

Long COVID is associated with substantial healthcare use, specialty referral, and multidisciplinary care needs (39, 40). A mechanism-informed trigger-timing approach may therefore have implications for healthcare delivery. Patients with Long COVID commonly present with multisystem symptoms that can generate multiple parallel referrals, repeated testing, and symptom-specific treatment pathways. A structured approach that identifies the most reproducible and clinically limiting physiological pattern could potentially help prioritize specialty referral, select confirmatory testing, and organize treatment sequencing. At a health-system level, standardized trigger-timing phenotypes may also assist in designing multidisciplinary pathways and identifying which patients require autonomic, rehabilitation, gastrointestinal, vascular, endocrine, or other targeted evaluation. These potential efficiencies remain hypothetical. Prospective implementation studies should determine whether mechanism-informed triage improves diagnostic yield, reduces unnecessary testing or referral duplication, shortens time to appropriate care, improves outcomes, or is cost-effective compared with conventional symptom-based pathways.

### 6.11 Clinical and research implications

Despite these limitations, the trigger-timing framework may provide a practical method for organizing complex symptom behavior in Long COVID. Its main contribution is not that it identifies a new biological mechanism, but that it offers a structured way to connect patient-reported symptom dynamics to mechanism-informed clinical reasoning.

By focusing on reproducible stress-response patterns, the framework may help clinicians prioritize testing and treatment sequencing, while helping researchers enrich cohorts for mechanism-specific studies. Its clinical and translational value will depend on whether future research demonstrates that trigger-timing patterns improve biological stratification, treatment matching, or outcome prediction beyond symptom-based classification alone.

## 7 Conclusion

This manuscript translates the companion network-disorder model into a clinical operating framework (29). Its central proposal is that reproducible trigger-timing behavior can help clinicians decide which physiological domains should be evaluated first.

The key inputs are trigger, timing, symptom pattern, and relief or recovery pattern. Interpreted together, they can support hypotheses about autonomic, bioenergetic/PEM, endothelial/microvascular, gastrointestinal, mast cell/histamine, neuroendocrine, or amplifier-dominant patterns without treating these domains as mutually exclusive or diagnostic categories.

The framework remains probabilistic and requires prospective validation. Its value will depend on whether trigger-timing groups correlate with objective measures, predict differential treatment response, and improve outcome tracking beyond symptom presence alone.

By focusing on observable stress-response behavior, the trigger-timing framework provides an operational bridge between biological stratification and practical clinical decision-making in Long COVID.
