# Supplementary material for: The trigger-timing framework for long COVID: using symptom triggers and temporal patterns to guide mechanistic stratification and treatment planning
Source: Front Med (Lausanne). 2026 Sep 16;13:1898440. doi: 10.3389/fmed.2026.1898440 (PMC13623576; doi:10.3389/fmed.2026.1898440)
Supplement: Supplementary file 1 [file Table1.docx]

**Supplementary Table 1. Expanded Trigger-Timing Assessment for Mechanism Identification in Long COVID.** This table provides an expanded operational framework for applying trigger-timing interpretation in Long COVID. It integrates trigger exposure, timing pattern, symptom expression, relief or recovery behavior, and mechanism-informed interpretation to support clinical hypothesis generation. These patterns are intentionally heuristic and non-exclusive. Individual patients may demonstrate overlapping, mixed, or evolving trigger-timing responses that span multiple physiological domains simultaneously. The table is intended to guide clinical reasoning and research enrichment rather than function as a deterministic diagnostic instrument.

| **Trigger** | **Timing Pattern** | **Symptom Pattern** | **Relief / Recovery Clue** | **Mechanism-Informed Interpretation** |
| --- | --- | --- | --- | --- |
| Standing / upright posture | Immediate | Tachycardia, dizziness, presyncope, upright brain fog, coat-hanger pain | Improves when supine | Orthostatic intolerance / POTS pattern |
| Standing / upright posture | Cumulative | Progressive fatigue, brain fog, heaviness with prolonged upright time | Improves with lying down, fluids, salt, compression | Hypovolemia, venous pooling, autonomic intolerance |
| Standing / upright posture | Same day | Heavy legs, exertional dyspnea, head pressure | Worsens with heat or activity | Autonomic plus endothelial / microvascular overlap |
| Heat exposure | Immediate | Tachycardia, dizziness, presyncope, weakness | Improves with cooling and lying down | POTS / orthostatic circulatory instability |
| Heat exposure | Immediate to short delay | Flushing, itching, hives, diarrhea, congestion, headache | Improves with trigger avoidance or antihistamine-type response | Mast cell / histamine-mediated pattern |
| Heat exposure | Progressive or same day | Heavy legs, head pressure, reduced stamina, dyspnea | Worse upright, worse with exertion | Endothelial / microvascular dysfunction |
| Cold exposure | Immediate | Tremor, palpitations, anxiety-like activation, internal vibrations | Improves with warming or calming | Sympathetic overactivation |
| Cold exposure | Persistent or delayed | Cold intolerance, slowed thinking, constipation, fatigue | Persistent baseline pattern | Thyroid or metabolic slowing pattern |
| Physical exertion | Immediate | Tachycardia, dizziness, dyspnea, head pressure | Improves with stopping or lying down | Autonomic or circulatory limitation |
| Physical exertion | Same day | Fatigue, muscle heaviness, reduced stamina without next-day crash | Improves with rest, but recovery is not prolonged | Non-PEM bioenergetic limitation or perfusion limitation |
| Physical exertion | Delayed 12 to 48 hours | Heavy limbs, flu-like malaise, sore throat, cognitive crash, worsened pain | Requires prolonged rest; worsens with repeated exertion | PEM / impaired bioenergetic recovery |
| Cognitive exertion | Immediate | Head pressure, overstimulation, palpitations, anxiety-like activation | Improves with stopping stimulation | Sympathetic activation or neurocognitive intolerance |
| Cognitive exertion | Delayed 12 to 48 hours | Cognitive crash, brain fog, flu-like malaise, sensory sensitivity | Requires prolonged cognitive rest | PEM-type cognitive crash |
| Meals in general | Immediate | Tachycardia, fatigue, dizziness, sleepiness | Smaller meals help; lying down may help | Postprandial autonomic stress / splanchnic pooling |
| Meals in general | Short delay | Fatigue, brain fog, shakiness, sleepiness | Improves with meal composition changes | Metabolic / glycemic dysregulation |
| Meals in general | Short delay | Flushing, itching, congestion, diarrhea, palpitations | Improves with food avoidance or antihistamine-type response | MCAS / histamine-mediated physiology |
| Carbohydrate-heavy meals | Short delay | Fatigue, shakiness, brain fog, sleepiness, head pressure | Improves with lower carbohydrate load | Glucose / insulin dysregulation or metabolic instability |
| Fermentable / high-fiber foods | Delayed | Bloating, gas, abdominal pain, reflux, diarrhea or constipation | Improves with trigger-food avoidance | Gut dysbiosis, fermentation pattern, gut-autonomic interaction |
| Alcohol | Immediate to short delay | Flushing, tachycardia, congestion, headache, insomnia | Improves with avoidance | Histamine / mast cell pattern, sleep destabilization |
| Emotional stress | Immediate | Palpitations, tremor, internal vibrations, chest tightness, insomnia | Improves with autonomic down-regulation | Sympathetic dominance / fight-or-flight pattern |
| Emotional stress | Delayed or same day | Fatigue, crash, brain fog, inflammatory-feeling flare | Requires rest; may worsen sleep | Neuroimmune or stress-axis amplification |
| Sleep deprivation | Next day | Brain fog, fatigue, orthostatic worsening, pain sensitivity | Improves with sleep stabilization | Sleep-related cross-domain amplifier |
| Nighttime / lying down | Circadian | Internal vibrations, insomnia, palpitations, hyperarousal | Worse at night | Sympathetic dominance, circadian dysregulation |
| Nighttime / lying down | Short delay | Reflux, bloating, cough, throat symptoms, disturbed sleep | Improves with reflux/gut management | Gut-autonomic or reflux-mediated pattern |
| Dehydration / fluid loss | Immediate or morning worse | Tachycardia, dizziness, brain fog, weakness | Improves with fluids, salt, compression | Hypovolemia / orthostatic intolerance |
| Intercurrent illness / infection | Same day to prolonged flare | Fatigue, pain, brain fog, autonomic worsening, PEM worsening | Recovery may take days or weeks | Immune activation / inflammatory amplification |
| Menstrual or hormonal shifts | Cyclic | Migraine, sleep disruption, mood changes, heat intolerance, fatigue | Predictable cycle relationship | Sex hormone / neuroendocrine modulation |

**Abbreviations:** MCAS, mast cell activation syndrome; PEM, post-exertional malaise; POTS, postural orthostatic tachycardia syndrome.
